# Tectorigenin alleviates the apoptosis and inflammation in spinal cord injury cell model through inhibiting insulin-like growth factor-binding protein 6

**DOI:** 10.1515/med-2023-0680

**Published:** 2023-04-11

**Authors:** Liqiang Zhou, Kui Yan, Shuxing Xing, Jun Cheng

**Affiliations:** Department of Orthopedics, Chengdu Fifth People’s Hospital, Chengdu, Sichuan Province, 611130, China; Department of Orthopedics, Chengdu Fifth People’s Hospital, No. 33 Mashi Street, Wenjiang District, Chengdu, Sichuan Province, 611130, China

**Keywords:** spinal cord injury, PC12, LPS, tectorigenin, IGFBP6

## Abstract

Since tectorigenin has been reported to possess anti-inflammation, redox balance restoration, and anti-apoptosis properties, we determine to unravel whether tectorigenin has potential in alleviating spinal cord injury (SCI). Herein, PC12 cells were induced by lipopolysaccharide (LPS) to establish *in vitro* SCI models. The cell viability and apoptosis were detected through cell counting kit-8 and flow cytometry assays. The caspase-3/8/9 content was measured by colorimetric method. Western blot was conducted to quantify the expressions of cleaved caspse-3/8/9, IGFBP6, TLR4, IκBα, p-IκBα, RELA proto-oncogene, p65, and p-p65. Enzyme-linked immunosorbent assay and real-time quantitative polymerase chain reaction were carried out to quantitate expressions of IGFBP6, interleukin-1β (IL-1β), interleukin-6 (IL-6), and tumor necrosis factor-α (TNF-α). SwissTargetPrediction and GSE21497 database were utilized to predict the potential therapeutic targets of tectorigenin. Comparison of IGFBP6 expression in SCI tissues and normal tissues was analyzed by GEO2R. Our study found that LPS induced the declined cell viability, elevated cell apoptosis, upregulation of caspase-3/8/9, cleaved caspase-3/8/9, IL-1β, IL-6, TNF-α, IGFBP6, and TLR4, and the activation of IκBα and p65 in PC12 cells. Tectorigenin reversed the above effects of LPS. IGFBP6 was predicted to be the potential therapeutic target of tectorigenin and was overexpressed in SCI tissues. Notably, IGFBP6 overexpression offset the effects of tectorigenin on PC12 cells. In conclusion, tectorigenin could alleviate the LPS-induced apoptosis, inflammation, and activation of NF-κB signaling in SCI cell models via inhibiting IGFBP6.

## 1 Introduction

Traumatic spinal cord injury (SCI) is a troublesome disease that not only seriously disturbs patients physically or mentally but also brings heavy burden [1,2]. SCI could be classified into primary and secondary phases [3,4]. The secondary SCI could occur after primary events and enlarge the lesion of neural tissue injury or aggravate the neurological deficits [5,6]. In this phase, the macrophages, microglia, T cells, and neutrophils could be recruited to form inflammatory infiltration and contribute to the disruption of blood–spinal cord barrier [2]. The release of interleukin-1β (IL-1β), interleukin-6 (IL-6), and tumor necrosis factor-α (TNF-α) is triggered, and the disrupted ionic homeostasis-associated cell apoptosis also occurred in the complex complications [7,8,9,10,11]. Hence, it can be concluded that anti-inflammation and anti-apoptosis are fundamental strategies in attenuating SCI.

After first aid and diagnosis, treatment should focus on patients’ life-threatening injuries and long-term complications, depending on the possibility of severe vascular tone or bradycardia triggered by the interruption of spinal cord sympathetic fibers [12]. Apart from newly examined pharmacological, non-pharmacological, or cell therapies, traditional steroids, such as methylprednisolone, riluzole, magnesium, and minocycline, are widely adopted to exert the effects of anti-inflammation, neuroprotection, wound healing, or excitoinhibition [13,14,15,16,17]. Despite the abundant therapeutic choices, more candidates still need to be explored, due to the possible medical contraindication of patients or the shortcomings of existing drugs.

Tectorigenin (TEC) is one of the flavonoids extracted from *Belamcanda chinensis*, which was suggested to be an antagonism of the activation of nuclear factor kappa B subunit 1 (NF-κB) signaling pathway to exert an anti-inflammatory effect [18]. TEC also plays protective roles in many organ injuries through suppressing inflammation or oxidation [19,20,21,22,23,24,25]. Notably, TEC could alleviate the lipopolysaccharide (LPS)-induced acute lung injury through promoting superoxidase dismutase activity and inhibiting myeloperoxidase activity, NF-κB activation, and neutrophil infiltration in lung tissues [19]. Its influences on improving hepatic failure are achieved via the declined serum contents of alanine aminotransferase and aspartate aminotransferase in mice and the inhibition of toll-like receptor 4 (TLR4)/MAPK or TLR4/NF-κB in macrophages [20]. The H_2_O_2_-induced oxidative stress injury in human umbilical vein endothelial cells could also be mitigated by TEC through rebalancing redox and inhibiting apoptosis [21]. As an anti-inflammation drug, dexamethasone also exerted cytotoxicity toward human airway epithelial cells, which could be relieved by TEC through promoting cell viability and inhibiting cell apoptosis [24]. Given the above information, it is worth exploring the exact role of TEC in SCI.

Moreover, TLR4 is one of the cell membrane receptors of LPS [26]. It could transmit signaling to myeloid differentiation factor 88 (MyD88) and then activate the downstream NF-κB or MAPKs [27]. Thus, TLR4/NF-κB signaling axis is the main inflammatory cascade involved in our researches for illuminating the functions of TEC in SCI.

## 2 Materials and methods

### 2.1 Drugs

Analytical grade LPS (L3024) was obtained from Sigma-Aldrich (St. Louis, MO, USA, https://www.sigmaaldrich.cn/CN/zh/product/sigma/l3024?context=product), which was dissolved in cell culture medium and stored at 2–8℃.

Analytical grade TEC (PHL80544, purity ≥95%) was also purchased from Sigma-Aldrich (St. Louis, MO, USA, https://www.sigmaaldrich.cn/CN/zh/product/supelco/phl80544?context=product).

### 2.2 Cell culture

Rat pheochromocytoma cell line PC12 (CRL-1721) was ordered from American Type Culture Collection (ATCC, Manassas, VA, USA). Cells were maintained in ATCC-formulated RPMI-1640 medium (30-2001, ATCC) supplemented with 10% fetal bovine serum (FBS; 10099, Gibco, Waltham, MA, USA) and 100 U/mL of penicillin and 100 μg/mL streptomycin (1% P/S, 15140122; Gibco) and cultivated in a 5% CO_2_ incubator (3110; Thermo Fisher Scientific, Waltham, MA, USA) at 37℃.

PC12 cells treated with 25, 50, 100, and 200 μM TEC for 12 h was used to detect corresponding cell viability [20]. Thereafter, PC12 cells used for subsequent experiments were treated with the selected concentrations of TEC (25, 50, and 100 μM) for 12 h and then induced by 5 μg/mL LPS for 12 h in LPS + TEC25/50/100 group. As a comparison, PC12 cells in the control group were cultured at the normal condition for 24 h. PC12 cells in the LPS + negative control (NC) group were transfected with empty vector, cultured under the normal condition for 12 h, and then stimulated with 5 μg/mL LPS for 12 h. PC12 cells in the LPS + TEC + IGFBP6/NC group were transfected with IGFBP6 overexpression vector or empty vector before treatment with 100 μM TEC for 12 h and the following 5 μg/mL LPS stimulation for 12 h. As a control, PC12 cells in the NC group were transfected with empty vector and cultured under the normal condition for 24 h [28].

### 2.3 Cell transfection

Overexpression plasmids for IGFBP6 were constructed by inserting whole coding sequence of IGFBP6 into pcDNA 3.1 empty vector (V79020, Invitrogen, Carlsbad, CA, USA). Empty vector was used in the NC group. Based on the provider’s procedure, PC12 cells were cultured overnight in advance. The next day, fresh 1640 medium without FBS was used to dilute Lipofectamine 2000 reagent (11668027, Invitrogen) and IGFBP6 overexpression plasmid (IGFBP6 group) or NC, respectively. Then, the two dilution reagents were mixed well and then stood for 5 min. Later, the mixture was added to the cells in each well, and after 4–6 h of incubation, the culture medium was replaced with complete medium. Cells were transfected for a total of 24 h.

### 2.4 Cell counting kit-8 (CCK-8) assay

CCK-8 assay reagent (CK04, Dojindo, Tokyo, Japan) was used to evaluate cell viability. Cells in 96-well plates (3,000 cells/well) were treated with 25, 50, 100, and 200 μM TEC for 12 h as mentioned above, and the experiments were repeated in triplicate. After that, 10 μL of CCK-8 solution was added into each well and cultivated in a 5% CO_2_ incubator at 37℃ for another 4 h. Finally, the OD value was read at 450 nm by a microplate reader (Sunrise, Tecan, Austria) to measure the viability of PC12 cells.

### 2.5 Enzyme-linked immunosorbent assay (ELISA)

The culture media of PC12 cells under diverse treatments mentioned above were collected and centrifuged at 500*g* for 5 min, and the supernatant was used for ELISA detection. IL-1β, IL-6, and TNF-α levels in the supernatant were measured using corresponding ELISA kits (MM-0047R1 for IL-1β, MM-0190R2 for IL-6, MM-0180R2 for TNF-α, Meimian, Jiangsu, China, http://www.mmbio.cn/). First, standard control reagents, washing buffer, and substrate solution were prepared in advance according to the instructions. Then, the prepared standard sample was diluted through adding calibrator buffer. The serial concentrations for IL-1β, IL-6, and TNF-α were diluted for establishing standard samples. The pure calibrator diluent was used as 0 pg/mL standard control. Then, pre-coated reaction well was taken out and fixed on plates.

The serial concentrations of standard IL-1β, IL-6, or TNF-α samples and pre-diluted samples to be tested were supplemented to a whole aliquot of 100 μL. The microplates were sealed with adhesive strips and incubated at room temperature (RT) for 2 h. Each well was washed using washing buffer five times and then patted until dry. Next, 100 μL of biotinylated IL-1β, IL-6, or TNF-α antibody was first seeded into each well, and 100 μL of horseradish peroxidase (HRP)-labeled streptavidin was then added. Following both steps above, the microplates were sealed and incubated at RT for 20 min in the dark, and the plates were patted dry. Later, 100 μL of TMB chromogenic reagent was added to each well, and the microplates were sealed and incubated at RT for 20 min in dark. Finally, 50 μL of termination buffer solution was added to each well, and all solutions were mixed through slight shaking. The OD value (450 nm) was detected using xMark Microplate absorbance spectrophotometer (# 1681150; Bio-Rad, CA, USA). The concentration of IL-1β, IL-6, or TNF-α was calculated according to the established standard curve.

### 2.6 Flow cytometry analysis

The apoptosis rate of PC12 cells with corresponding treatment in each group was detected using Annexin V-FITC/propidium iodide (PI) Apoptosis Detection Kit (C1062L; Beyotime). Following the manufacturer’s guideline, adherent PC12 cells were digested using trypsin (C0202; Beyotime) and rinsed off. Then, cells were collected, centrifuged, and re-suspended using phosphate-buffered saline (PBS). Cell number was counted. About 5 × 10^4^ re-suspended PC12 cells were centrifuged. The precipitate was re-suspended again using 195 μL of Annexin V-FITC binding buffer. Then, the re-suspended cells were added with 5 μL of Annexin V-FITC and 10 μL of PI and incubated in the dark at RT for 15 min. Cell apoptosis was detected by flow cytometry instrument (BD Biosciences), and the data were analyzed with FlowJo software (Stanford University, San Francisco, CA, USA).

### 2.7 Colorimetric detection for caspase 3/8/9

The colorimetric kits for caspase-3 (KGA204), caspase-8 (KGA304), and caspase-9 (KGA404) were purchased from Nanjing KeyGen Biotech (Nanjing, China). After cells were lysed using radio immunoprecipitation assay (RIPA) lysis buffer (P0013B; Beyotime), the lysate was applied to detect the activity of caspases mentioned above according to their individual specification. Following incubation with their specific reaction buffer at 37℃ for 4 h in the dark, the results were counted through measuring absorbance (405 nm) with Sunrise microplate reader [29].

### 2.8 Bioinformatic analysis

SwissTargetPrediction (http://www.swisstargetprediction.ch/) and GEO2R (https://www.ncbi.nlm.nih.gov/geo/geo2r/) were applied to predict the potential effectors of TEC in disease treatment. GEO2R was also used to analyze the expression level of IGFBP6 in SCI tissues, when compared with that in normal tissues using GSE21497 database.

### 2.9 Real-time quantitative polymerase chain reaction (RT-qPCR) assay

Total RNA of PC12 cells treated as mentioned above was isolated using TRIzol Reagent (15596026; Invitrogen) and reversely transcribed into cDNA by reverse transcription reagents (AH401-01; TransGen, Beijing, China). Next, SYBR Green Real-Time PCR Master Mix (AQ301-01; TransGen) and primers were used to detect the gene expression using CFX Opus Real-time PCR system (CFX96; Bio-Rad). The results were calculated by 2^−ΔΔCt^ method [30] with glyceraldehyde-3-phosphate dehydrogenase (GAPDH) as the internal reference. The primers of IL-1β, IL-6, TNF-α, IGFBP6, and GAPDH were as follows: IL-1β, 5′-GGCTGACAGACCCCAAAAGA-3′ (forward), 5′-TGTCGAGATGCTGCTGTGAG-3′ (reverse); IL-6, 5′-AGAGACTTCCAGCCAGTTGC-3′ (forward), 5′-AGTCTCCTCTCCGGACTTGT-3′ (reverse); TNF-α, 5′-ATGGGCTCCCTCTCATCAGT-3′ (forward), 5′-GCTTGGTGGTTTGCTACGAC-3′ (reverse); IGFBP6, 5′-CCAAGGAGAGCAAACCCCAT-3′ (forward), 5′-CTTGAACAGGACTGGGCCTT-3′ (reverse); and GAPDH, 5′-CACCATCTTCCAGGAGCGAG-3′ (forward), 5′-GACTCCACGACGTACTCAGC-3′ (reverse).

### 2.10 Western blot (WB) assay

Total protein in PC12 cells treated as mentioned above was extracted through RIPA lysis buffer supplemented with protease and phosphatase inhibitor (P1051; Beyotime) and 0.1 mM phenylmethylsulfonyl fluoride (ST506; Beyotime). BCA protein assay kit (P0010; Beyotime) was applied to detect protein concentration. All protein samples to be used in one experiment were diluted to the same concentration. Then, an equal number of proteins were subjected to sodium dodecyl sulfate-polyacrylamide gel electrophoresis (P0012A; Beyotime) and transferred to polyvinylidene fluoride (PVDF) membrane (IPSN07852; Millipore, Bedford, MA, USA). PVDF membranes were subsequently blocked with 5% nonfat milk for 1 h and then cut into strips containing different protein bands according to the protein marker.

Strips were incubated with primary antibodies for IGFBP6 (bs-4064R, rabbit polyclonal, 23 kDa, dilution 1:500; Bioss, MA, USA), cleaved caspase-3 (# 9661, rabbit antibody, 17 and 19 kDa, dilution 1:1,000; Cell signaling technology, Danvers, MA, USA), cleaved caspase-8 (#98134 rabbit antibody, 41 kDa, dilution 1:1,000; Cell signaling technology), cleaved caspase-9 (# 9507, rabbit antibody, 17 and 38 kDa, dilution 1:1,000; Cell Signaling Technology), caspase-3 (#9662, rabbit antibody, 30 kDa, dilution 1:1000, Cell signaling technology), caspase-8 (#4927, rabbit antibody, 57 kDa, dilution 1:1000, Cell signaling technology), caspase-9 (#9504, rabbit antibody, 49 kDa, dilution 1:1000, Cell signaling technology), TLR4 (ab13867, rabbit polyclonal, 90 kDa, dilution 1:500; Abcam, Cambridge, UK), p-IκBα (# 2859, rabbit monoclonal, phospho-Ser32, 40 kDa, dilution 1:1,000; Cell Signaling Technology), IκBα (# 4814, mouse monoclonal, 39 kDa, dilution 1:1,000; Cell Signaling Technology), p-p65 (# 3033, rabbit monoclonal, phospho-Ser536, 65 kDa, dilution 1:1,000; Cell Signaling Technology), p65 (# 8242, rabbit monoclonal, 65 kDa, dilution 1:1,000; Cell Signaling Technology), and GAPDH (ab181602, rabbit monoclonal, 36 kDa, dilution 1:10,000; Abcam) at 4℃ overnight. Then, the strips were washed by 0.1 M TBS containing 0.1% Tween-20 (ST673; Beyotime) three times, followed by incubation with HRP-conjugated secondary antibodies (ab205718, ab205719, dilution 1:5,000; Abcam) for 2 h at RT. Specific protein signals were tested using UltraSignal luminol-based enhanced chemiluminescence Western Blotting Substrate (4AW011-200; 4A Biotech, Beijing, China) and quantified using the ImageJ software (Wayne Rasband, NIH, USA).

### 2.11 Statistical analysis

All data were analyzed by Graphpad Prism 8.0 (GraphPad Software Inc, La Jolla, CA, USA), and the results were exhibited as mean ± standard deviation (SD). The significance of differences among multiple groups was analyzed using one-way analysis of variance. Tukey’s multiple comparison test was carried out for post hoc test. The data with *P* < 0.05 were considered statistical significant.

## 3 Results

### 3.1 LPS inhibited the viability and promoted the apoptosis of PC12 cells, while TEC could reverse the effects of LPS

**Figure 1:**
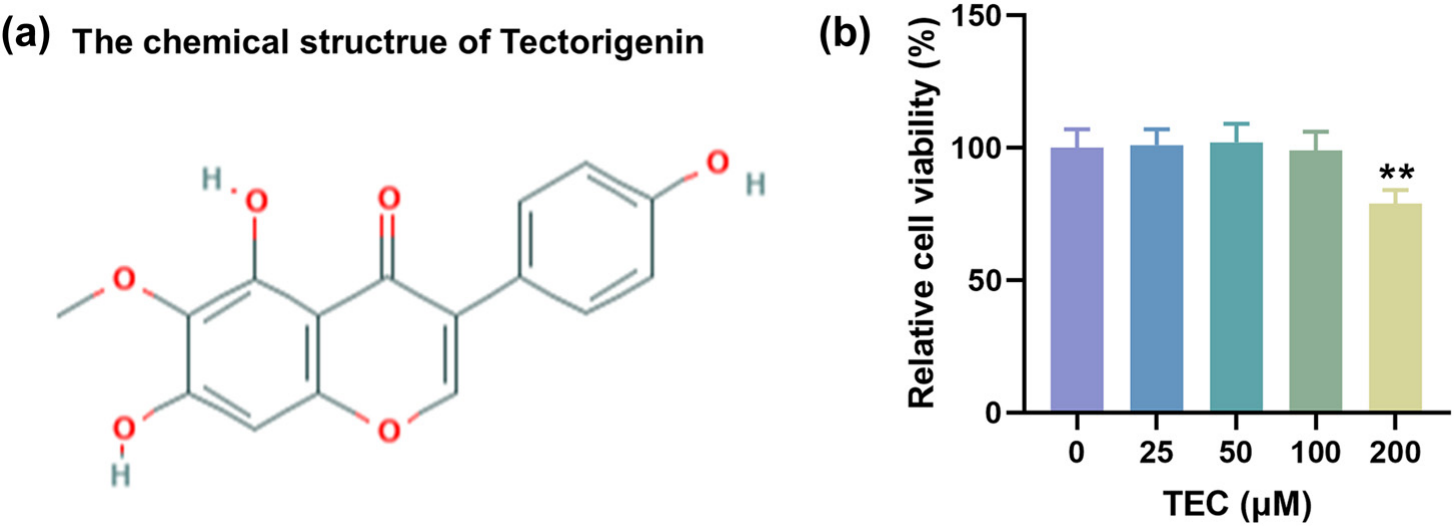
The concentration of TEC that was non-toxic to PC12 cells was detected. (a) The chemical structure of tectorigenin was presented. (b) CCK-8 assay was conducted to detect the viability of PC12 cells under the treatment of 0, 25, 50, 100, and 200 μM TEC. ^**^ *P* < 0.01 vs 0 μM TEC group. Results were described as means ± SD of triplicate determination.

The chemical structure of TEC is presented in Figure 1a. First, we detected the toxic effect of TEC on the PC12 cells. Through treating cells with 25, 50, 100, and 200 μM TEC, we found that only 200 μM TEC exerted slight cytotoxicity toward PC12 cells (Figure 1b, *P* < 0.01). Accordingly, 25, 50, and 100 μM TEC were adopted in the following experiments.

**Figure 2:**
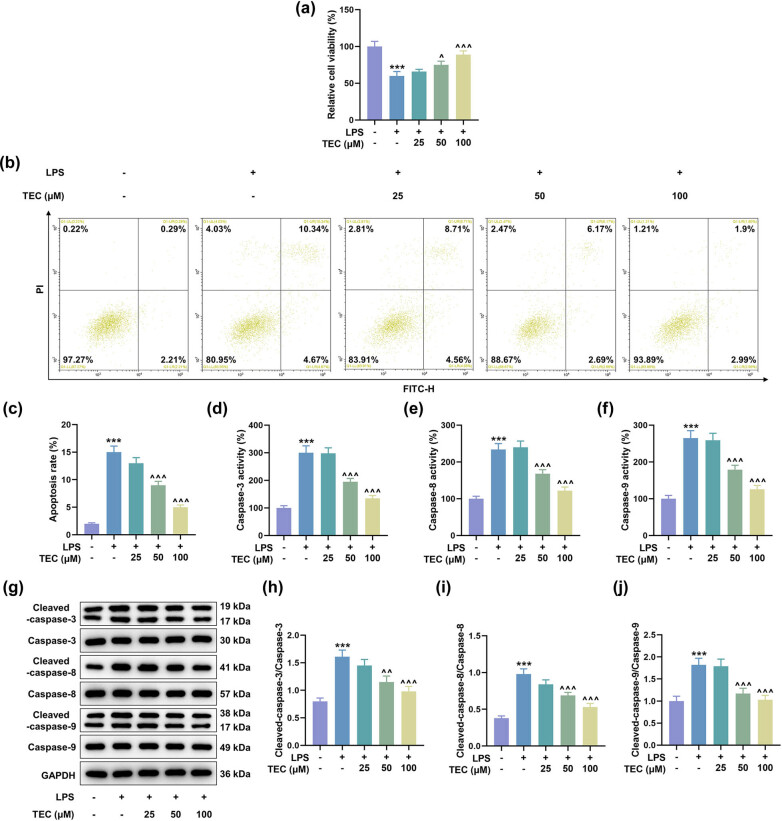
TEC alleviated the LPS-induced PC12 cellular injuries at a dose-dependent manner. (a) CCK-8 assay was applied to determine the viability of PC12 cells under the treatment of 5 μg/mL LPS coupled with or without treatment of 25, 50, or 100 μM TEC. ^***^ *P* < 0.001 vs control group, ^^^ *P* < 0.05 vs LPS group, ^^^^^ *P* < 0.001 vs LPS group. (b and c) Flow cytometry was performed to detect the apoptotic rate of PC12 cells under the treatment of 5 μg/mL LPS coupled with or without treatment of 25, 50, or 100 μM TEC. ^***^ *P* < 0.001 vs control group, ^^^^^ *P* < 0.001 vs LPS group. (d–f) Colorimetric assay was conducted to measure the expressions of apoptosis-related factors including caspase-3, caspase-8, and caspase-9. ^***^ *P* < 0.001 vs control group, ^^^^^ *P* < 0.001 vs LPS group. (g–j) WB assay was utilized to quantitate the expressions of apoptosis-related factors including caspase-3, caspase-8, caspase-9, cleaved caspase-3, cleaved caspase-8, and cleaved caspase-9. ^***^ *P* < 0.001 vs control group, ^^^^ *P* < 0.01 vs LPS group, ^^^^^ *P* < 0.001 vs LPS group. GAPDH was used as the internal reference for WB assay. All results in this figure were described as means ± SD of triplicate determination.

Then, PC12 cells were treated with 5 μg/mL of LPS to mimic the SCI model *in vitro*. The effect of TEC on the LPS-induced PC12 cells was determined. The results showed that LPS treatment inhibited the viability of PC12 cells, and TEC at 50 and 100 μM concentrations could reverse the inhibiting effect of LPS (Figure 2a, *P* < 0.05). In addition, LPS treatment promoted the apoptosis, the activity of caspase-3, caspase-8, and caspase-9, and the expressions of cleaved caspase-3, cleaved caspase-8, and cleaved caspase-9 (Figure 2b–j, *P* < 0.001). However, TEC (50 and 100 μM) could attenuate the LPS-induced elevation of PC12 cell apoptosis and the corresponding apoptosis-related factors including cleaved caspase-3, cleaved caspase-8, and cleaved caspase-9, as well as their concomitant activation forms (Figure 2b–j, *P* < 0.01).

### 3.2 TEC alleviated the promoting effect of LPS on the inflammatory factors in PC12 cells

**Figure 3:**
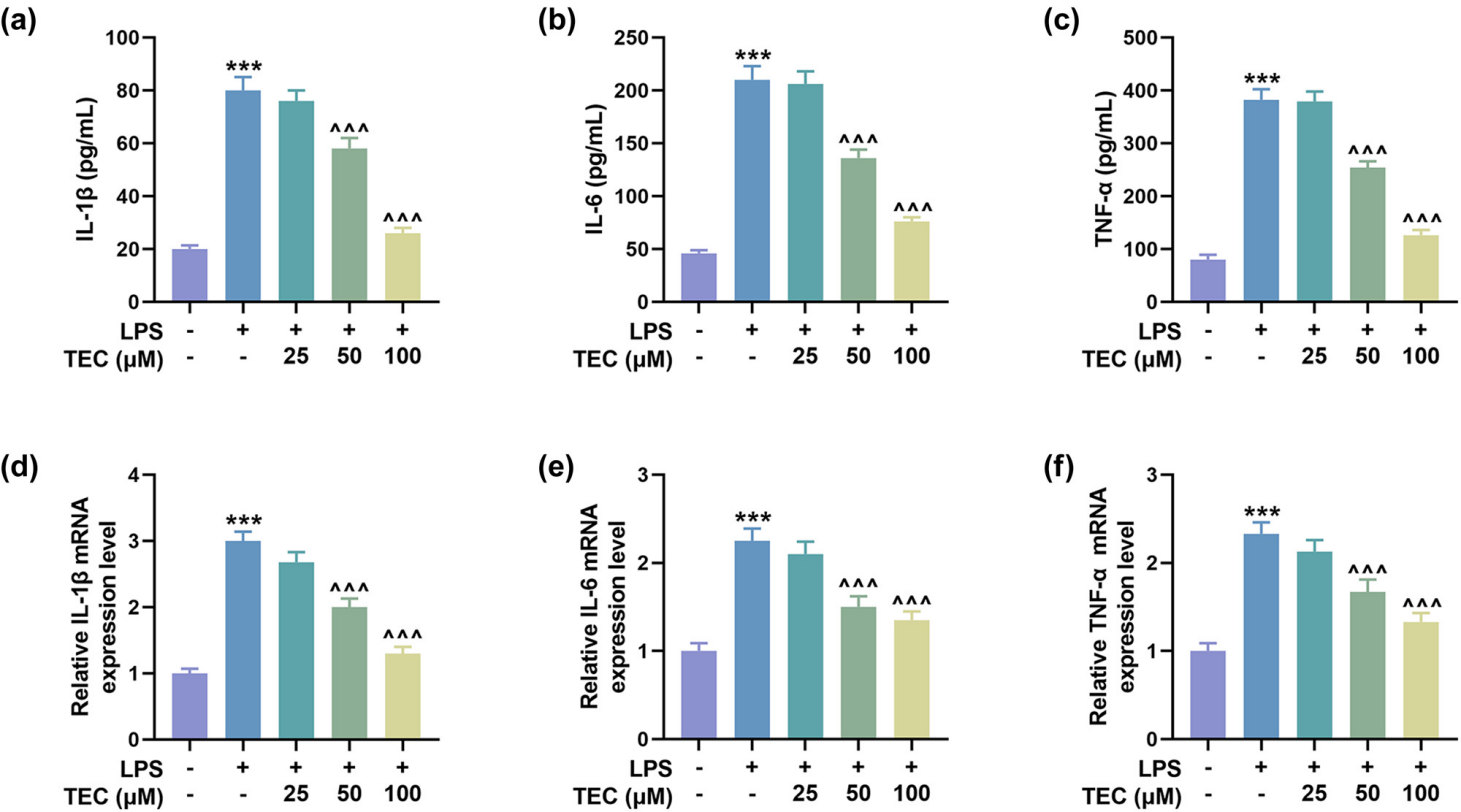
TEC abrogated the LPS-induced up-regulation of pro-inflammatory factors in PC12 cells. (a–c) ELISA was used to measure the levels of pro-inflammatory factors, including IL-1β, IL-6, and TNF-α, under LPS inducement with or without treatment of 25, 50, or 100 μM TEC. ^***^ *P* < 0.001 vs control group, ^^^^^ *P* < 0.001 vs LPS group. (d–f) RT-qPCR was performed to quantitate the expressions of pro-inflammatory factors including, IL-1β, IL-6, and TNF-α, under various treatments. ^***^ *P* < 0.001 vs control group, ^^^^^ *P* < 0.001 vs LPS group. GAPDH was used as the internal reference for RT-qPCR assay. All results in this figure were described as means ± SD of triplicate determination.

Controlling the inflammatory response is a critical step in the treatment of SCI. The expressions of pro-inflammatory factors, including IL-1β, IL-6, and TNF-α, were found to be up-regulated under LPS inducement and then were diminished by TEC (50 and 100 μM) in LPS-induced PC12 cells (Figure 3a–f, *P* < 0.001).

### 3.3 TEC inhibited IGFBP6 expression in LPS-induced PC12 cells

**Figure 4:**
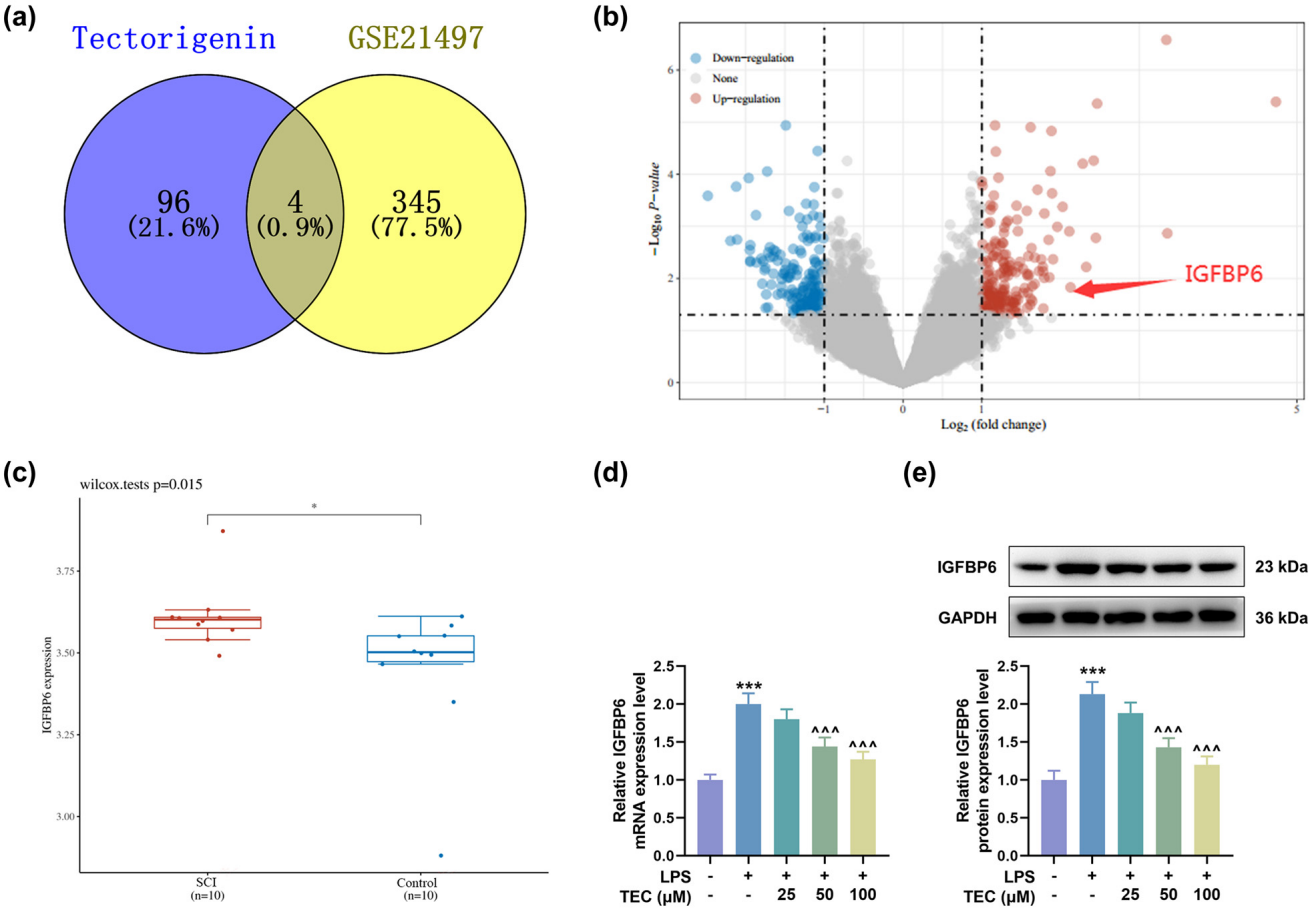
The expression of IGFBP6 was predicted and validated to be induced by LPS and inhibited by TEC. (a) SwissTargetPrediction (http://www.swisstargetprediction.ch/) and GSE21497 database were used to predict the potential target of TEC in disease treatment, and four targets were found, including TBXAS1, IGFBP6, OPRD1, and CA3. (b and c) GEO2R (https://www.ncbi.nlm.nih.gov/geo/geo2r/) was applied to analyze GSE21497 database, thereby obtaining the results that IGFBP6 was overexpressed in SCI tissues. (d and e) RT-qPCR and WB assays were carried out to measure the expression of IGFBP6 in PC12 cells under LPS inducement with or without the treatment of 25, 50, or 100 μM TEC. ^***^ *P* < 0.001 vs control group, ^^^^^ *P* < 0.001 vs LPS group. GAPDH was used as the internal reference for RT-qPCR and WB assays. All results in this figure were described as means ± SD of triplicate determination.

To unveil the associated downstream effector of TEC in SCI cell models, we used SwissTargetPrediction and GSE21497 to predict the potential target of TEC in disease treatment, and four targets were found, including TBXAS1, IGFBP6, OPRD1, and CA3 (Figure 4a). Since IGFBP6 was reported to aggravate SCI [31], we chose IGFBP6 as the research target in subsequent experiments. GEO2R was applied to analyze GSE21497 database, which revealed that IGFBP6 was overexpressed in SCI tissues (Figure 4b and c, *P* < 0.05). We then discovered that the expression of IGFBP6 was elevated in LPS-induced PC12 cells but then was dwindled by TEC treatment at a dose-dependent manner (Figure 4d and e, *P* < 0.05).

### 3.4 IGFBP6 overexpression attenuated the effects of TEC on the apoptosis of LPS-induced PC12 cells

**Figure 5:**
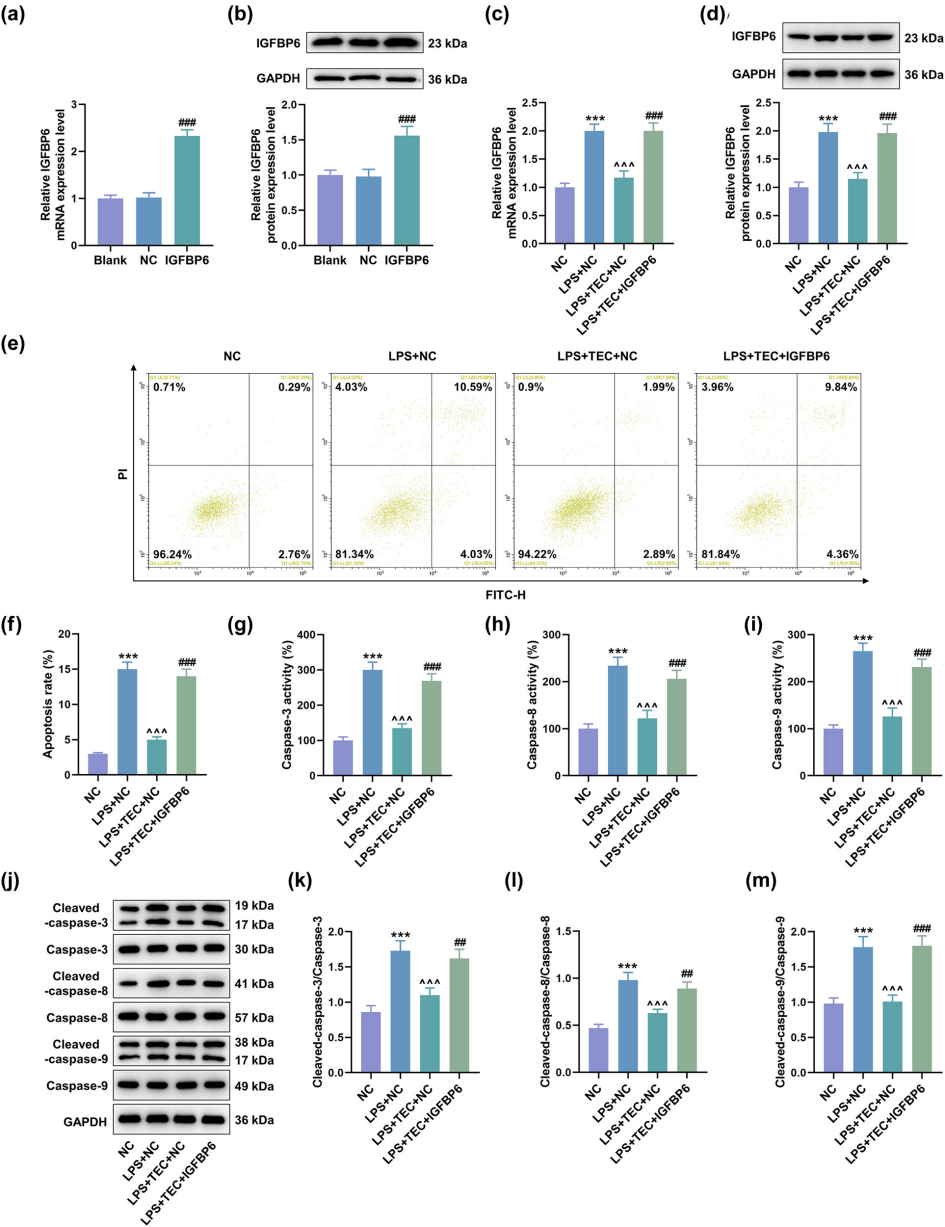
Overexpressed IGFBP6 reversed the inhibiting role of TEC in the apoptosis of LPS-induced PC12 cells. (a and b) RT-qPCR and WB assays were performed to detect the expression of IGFBP6 in PC12 cells with or without transfection of IGFBP6 overexpression plasmid (IGFBP6 group). ^###^ *P* < 0.001 vs. negative control (NC) group. (c and d) RT-qPCR and WB assays were applied to quantify the expression of IGFBP6 in PC12 cells with or without LPS inducement coupled with the treatment of NC, TEC + NC, or TEC + IGFBP6 overexpression plasmid. ^***^ *P* < 0.001 vs NC group, ^^^^^ *P* < 0.001 vs LPS + NC group, ^###^ *P* < 0.001 vs LPS + TEC + NC group. (e and f) Flow cytometry was performed to detect the apoptotic degree of PC12 cells with or without LPS inducement coupled with the treatment of NC, TEC + NC, or TEC + IGFBP6 overexpression plasmid. ^***^ *P* < 0.001 vs NC group, ^^^^^ *P* < 0.001 vs LPS + NC group, and ^###^ *P* < 0.001 vs LPS + TEC + NC group. (g–i) Colorimetric method was used to quantitate the apoptosis-related factors, including caspase-3, caspase-8, and caspase-9, that were released from PC12 cells with or without LPS inducement coupled with the treatment of NC, TEC + NC, or TEC + IGFBP6 overexpression plasmid. ^***^ *P* < 0.001 vs NC group, ^^^^^ *P* < 0.001 vs LPS + NC group, and ^###^ *P* < 0.001 vs LPS + TEC + NC group. (j–m) WB assay was utilized to determine the expressions of apoptosis-related factors, including caspase-3, caspase-8, caspase-9, cleaved caspase-3, cleaved caspase-8, and cleaved caspase-9, in PC12 cells under various conditions. ^***^ *P* < 0.001 vs NC group, ^^^^^ *P* < 0.001 vs LPS + NC group, and ^###^ *P* < 0.001 vs LPS + TEC + NC group. GAPDH was used as the internal reference for RT-qPCR and WB assays. All results in this figure were described as mean ± SD of triplicate determination.

To investigate whether TEC plays a role in SCI by regulating IGFBP6, PC12 cells were transfected with IGFBP6 overexpression plasmids, and the up-regulation of IGFBP6 in PC12 cells indicated the successful transfection (Figure 5a and b, *P* < 0.001). As depicted in Figure 5c and d, IGFBP6 overexpression reversed the declined IGFBP6 expressions in PC12 cells under TEC treatment (*P* < 0.01). TEC treatment could significantly offset the effects of LPS on the apoptosis and apoptosis-related factor expressions in PC12 cells. We then discovered that IGFBP6 overexpression reversed the inhibiting effect of TEC on apoptosis, activity of caspase-3, caspase-8, and caspase-9, and expressions of cleaved caspase-3, cleaved caspase-8, and cleaved caspase-9 in LPS-induced PC12 cells (Figure 5e–m, *P* < 0.001).

### 3.5 IGFBP6 overexpression counteracted the effects of TEC on the inflammation and TLR4/NF-κB pathway in LPS-induced PC12 cells

**Figure 6:**
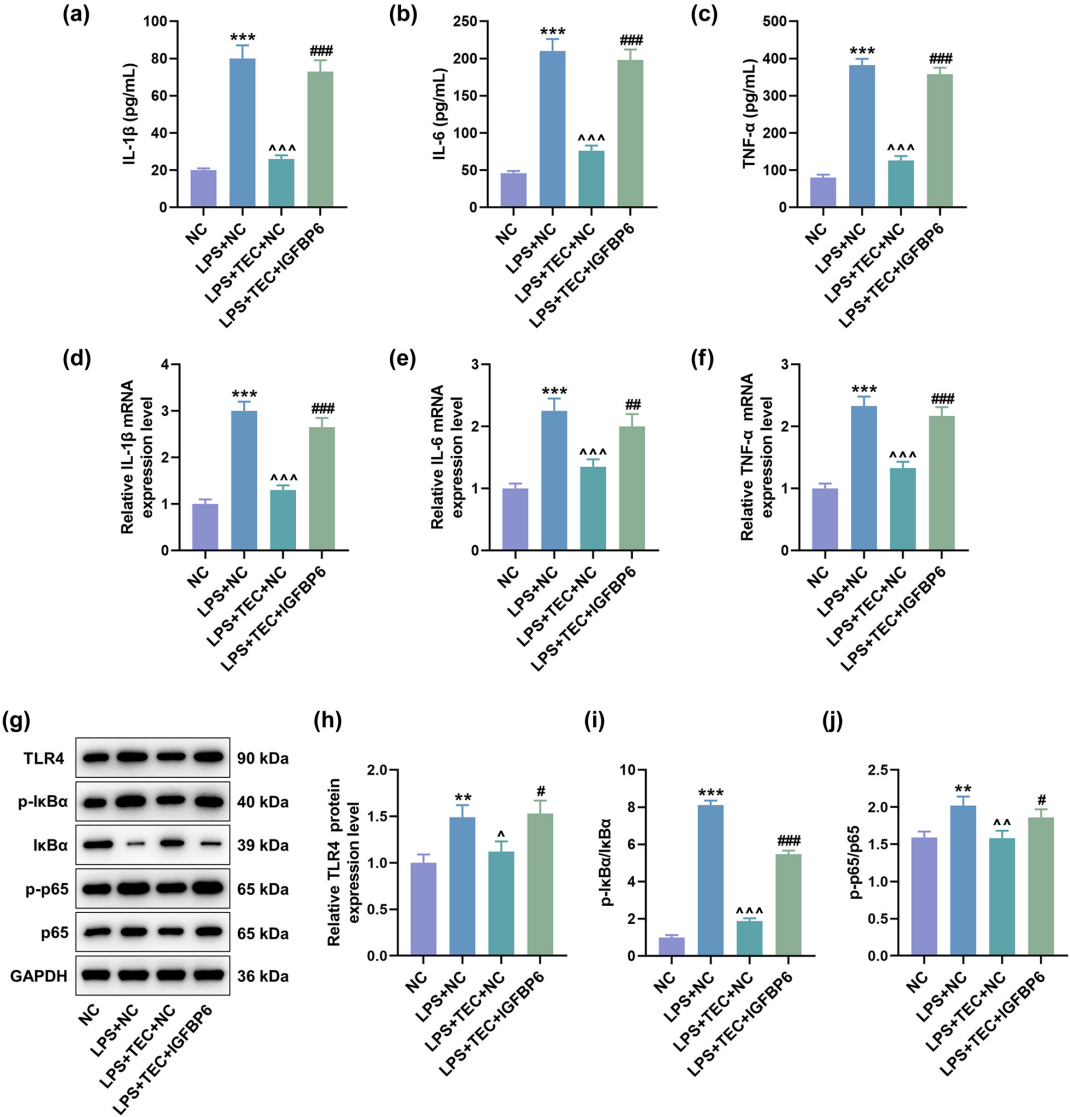
Overexpressed IGFBP6 offset the suppressing effect of TEC on inflammation in LPS-induced PC12 cells. (a–c) ELISA was used to measure the levels of pro-inflammatory factors, including IL-1β, IL-6, and TNF-α, in PC12 cells with or without LPS inducement coupled with the treatment of NC, TEC + NC, or TEC + IGFBP6 overexpression plasmid. ^***^ *P* < 0.001 vs NC group, ^^^^^ *P* < 0.001 vs LPS + NC group, and ^###^ *P* < 0.001 vs LPS + TEC + NC group. (d–f) RT-qPCR assay was exploited to detect the levels of pro-inflammatory factors, including IL-1β, IL-6, and TNF-α, in PC12 cells under various treatments. ^***^ *P* < 0.001 vs NC group, ^^^^^ *P* < 0.001 vs LPS + NC group, ^##^ *P* < 0.01 vs LPS + TEC + NC group, and ^###^ *P* < 0.001 vs LPS + TEC + NC group. (g–j) WB assay was performed to determine the expressions of TLR4/NF-κB pathway-related factors, including TLR4, IκBα, p-IκBα, p65, and p-p65, in PC12 cells under various conditions. ^**^ *P* < 0.01 vs NC group, ^***^ *P* < 0.001 vs NC group, ^^^^ *P* < 0.01 vs LPS + NC group, ^#^ *P* < 0.05 vs LPS + TEC + NC group, and ^##^ *P* < 0.01 vs LPS + TEC + NC group. GAPDH was used as the internal reference for RT-qPCR and WB assays. All results in this figure were described as mean ± SD of triplicate determination.

The effect of IGFBP6 on the pro-inflammatory factors was also detected. The results proved that IGFBP6 overexpression reversed the effects of TEC on down-regulating expressions of pro-inflammatory factors, including IL-1β, IL-6, and TNF-α (Figure 6a–f, *P* < 0.01). The regulatory effect of TEC on inflammatory response in diseases is realized by inhibiting TLR4/NF-κB signaling pathway [20], which is also related to the expression of IGFBP6 [32]. The results demonstrated that LPS induced the activation of TLR4/NF-κB signaling pathway, as evidenced by the up-regulation of TLR4, p-IκBα/IκBα, and p-p65/p-65 (Figure 6g–j, *P* < 0.01). TEC treatment inhibited the activation of TLR4/NF-κB signaling pathway, while the function of TEC was offset by IGFBP6 overexpression (Figure 6g–j, *P* < 0.05).

## 4 Discussion

Through analyzing all experimental results, it can be concluded that TEC could exert neuroprotective effects toward inflammatory infiltration of neurons and reduce the associated cellular apoptosis. Notably, all these functions were realized through inhibiting downstream apoptotic or inflammatory effector, IGFBP6.

The anti-inflammation or anti-apoptosis effect of TEC was not only validated in our experiments but also reviewed in our introduction section. Therefore, TEC is actually beneficial to mitigate SCI especially in the second phase that is full of inflammation infiltration and accompanied with various complications. Some similar or different neuroprotective effects of TEC have also been reported as follows [33,34,35]. TEC could modulate the expression of erythropoietin through inhibiting the degradation of hypoxia-inducible factor-1α (HIF-1α), therefore exerting neuroprotective functions on *in vitro* cultured neuron-like NT2/D1 cells and rat cortical neurons [33]. TEC could also inhibit TLR4/MyD88/NF-κB and ERK/JNK signaling pathways in microglial cells that are over-activated under the treatment of LPS [34]. The overproduction of relative inflammatory mediators including, NO synthase, cycloxygenase-2, TNF-α, and IL-6, are also attenuated through TEC treatment [34]. Accordingly, the neuroprotective role of TEC is out of question.

We then strictly carried out experiments to identify whether IGFBP6 can promote apoptosis or inflammation, since its expression was suppressed by TEC. The results confirmed that IGFBP6 could activate apoptotic cascade, pro-inflammatory reactions, and the NF-κB signaling. According to former findings, IGFBP6 has been examined in diverse situations and these results are controversial [36,37,38,39,40,41,42,43,44,45,46]. Several literatures reported that IGFBP6 is the predominant IGFBP synthesized by PC12 pheochromocytoma [36,37,38]. A review proposed that IGFBP6 plays an important role in the control of cell-specific immunologic adaptation following hyperthermia treatment [39]. A simultaneously published article elucidated that IGFBP6 could induce the oxidative burst, degranulation and chemotaxis of neutrophils [40]. A similar report also confirmed the chemotactic role of IGFBP6 in rheumatoid arthritis [41]. Moreover, the content of IGFBP6 could be lessened through the treatment of dexamethasone, an anti-inflammation drug [38,41]. IGFBP6 has been demonstrated to be positively correlated with NF-κB signaling or other inflammatory factors, including IL-6 and IL-17B [42,43].

In addition to the aforementioned report revealing IGFBP6 as a pro-inflammatory effector, some contrary findings have been published [44,45,46]. IGFBP6 has been verified to be associated with anti-inflammatory factor TGF-β1 and enhance epithelial-mesenchymal transition in fibroblasts to promote the recovery of skin injuries under the treatment of ozone oil [44]. The adipose stem cell-derived extracellular vesicles, which have been identified to suppress allergic airway inflammation, could promote the expression of IGFBP6 [45]. Furthermore, IGFBP6 has a negative relationship with IL-6 or IL-1β, since neonatal astrocytes induced by external injuries could produce IL-6 or IL-1β to facilitate neurogenesis, while IGFBP6 inhibits the differentiation of multipotent neural stem/progenitor cells to block neurogenesis [46]. Apart from pro-inflammatory effects, the pro-apoptotic role of IGFBP6 is also controversial [31,47]. IGFBP6 expression is upregulated after the establishment of SCI model, and IGFBP6 is positively correlated with the expressions of pro-apoptotic p53 and cleaved caspase-3 [31]. IGFBP6 derived from human bone marrow mesenchymal stem cells exerts neuroprotective effects through inhibiting the activation of Bax and promoting the phosphorylation of Akt [47].

## 5 Conclusions

Collectively, TEC might be an efficient substance that could repair the peripheral nervous system after exogenous incidence causes SCI through maintaining neural cell numbers and functions. Targeting IGFBP6 also seems to be useful in the recovery of SCI. The precision and complexity of nervous systems are well known, leading to controversial conclusions in previous reports. Hence, more precise results should be provided in future validation.
